# Practice towards Prevention and Control Measures of Coronavirus Disease and Associated Factors among Healthcare Workers in the Health Facilities of the Horo Guduru Wollega Zone, West Ethiopia, 2021

**DOI:** 10.1155/2022/1973502

**Published:** 2022-06-28

**Authors:** Atoma Negera, Chernet Hailu, Addis Birhanu

**Affiliations:** ^1^Nursing Department, College of Health Science, Mettu University, Metu, Ethiopia; ^2^Epidemiology Department, College of Public Health, Institute of Health, Jimma University, Jimma, Ethiopia

## Abstract

**Background:**

A novel coronavirus, a virus that causes coronavirus disease (COVID-19), was first identified in Wuhan, China, on December 2019. The virus affects the respiratory system and it is highly contagious, spreading from person to person. Healthcare workers are more at risk due to the nature of their work, which is caring for both COVID-19-affected and nonaffected patients. Lack of knowledge about the disease directly affects early diagnosis and treatment, which may result in the rapid spread of the infection in the community. Having enough knowledge about a disease can always affect an individual's attitudes and practices. However, there is limited evidence on the knowledge, attitude, practice of prevention, and control measures of COVID-19 and associated factors among healthcare workers (HCWs) in resource-limited countries, including Ethiopia.

**Methods:**

A facility-based cross-sectional study design was used among 334 samples of health workers who were selected using a stratified two-stage sampling technique, from health facilities of the Horo Guduru Wollega Zone from May to June 2021. A structured self-administered questionnaire was used to collect the data from the HCWs. The information collected was entered to EpiData version 3.1 and exported to SPSS version 21 software for further analyses. Bivariable and multivariable binary logistic regression analyses were used to identify factors associated with the KA practice of the HCWs. Those variables with a *p* value <05 with a 95% confidence interval (CI) were considered as statistically significantly associated with the outcome variable.

**Result:**

Among the participating HCWs, 208 (64%; 95% CI: (58.8%, 69.2%)) of them had good practices of prevention and control measures of COVID-19 with the mean (±SD) practice score was 7.63 ± 2.45. Multivariable binary logistic regression revealed that being a health center worker (AOR = 0.34, 95% CI: (0.19, 0.60)), being trained (AOR = 0.41, 95% CI: (0.21, 0 .82)), and having sufficient knowledge (AOR = 2.73, 95% CI: (1.35, 5.53)) were significantly associated with good preventive practice.

**Conclusion:**

The overall magnitude of practice of prevention and control measures of COVID-19 was not sufficient. Therefore, strategies for enhancing the capacity of healthcare workers to exercise practices of prevention and control measures of COVID-19 are needed.

## 1. Introduction

A novel coronavirus, a virus that causes coronavirus disease 19 (COVID-19), was first identified in Wuhan, China. The virus affects the respiratory system and it is highly contagious, spreading from person to person [1]. The World Health Organization (WHO) declared the coronavirus (COVID-19) as a pandemic disease on March 11th 2020 [2]. As of 06 February 2021, the virus affected 220 countries around the world and it had been estimated that about 104 million people were infected with COVID-19 worldwide, of which about 2.29 million have lost their precious life [3, 4]. The disease is extremely severe in higher age groups, smokers, and patients with exquisite preexisting medical conditions, such as cardiovascular diseases, chronic respiratory diseases, diabetes, and hypertension [5]. The incubation period of the virus ranges from 2 to 14 days, a mean of around 5 days. Thus, the recommended length of isolation is a minimum of 14 days [6]. There is some diversity in the initial symptoms. However, most COVID-19 patients had fever and mild to severe respiratory symptoms. History of visit to epidemic areas is vital and included in a case definition to accurately diagnose it. If an individual is suspected for COVID-19, well-timed referral to the public health authorities for testing is crucial [1, 7].

Healthcare workers are experiencing significant physical as well as psychological risks while working during COVID-19. All employed healthcare workers (HCWs) have encountered unexpected challenges in caring for patients [8]. They are frontline of the disease response and are exposed to dangers like pathogen exposure, occupational burnout and stigma, long working hours, fatigue, and physical violence [9]. Healthcare workers infected by the coronavirus report psychological problems like: stress, depressive symptoms, anxiety symptoms, and burnout as a result of COVID-19 [10, 11]. In the past few months, over a hundred thousand healthcare workers have lost their lives due to COVID-19, a tragedy to the world as they are a barrier to fight against the virus [12]. There is no evidence that reveals the exact number of HCWs infected with the COVID-19 virus. Some studies show 15% to 18% and in some cases up to 20% of HCWs were infected with COVID-19. According to Pan-American Health Organization (PAHO)), COVID-19 has infected more than 570, 000 healthcare workers and killed about 2,500 in the Americas, as on September 2, 2020 [13]. Among the factors that contribute to the infection of HCWs, the major ones are:—inadequate use and availability of Personal Protective Equipment (PPE), lack of understanding of the disease, uncertain diagnostic criteria, unavailability of diagnostic tests, and psychological stress. Therefore, the authorities should protect healthcare providers via education and training, the readiness of staff, incentives, the availability of PPEs, and psychological support [14].

The WHO has developed guidelines for healthcare workers and online refresher courses; the Center for Disease Control and various governmental and nongovernmental organizations in various countries are trying to boost the knowledge, attitude, and prevention strategies of healthcare workers [15]. Delay in early detection of cases and lack of adequate personal protective equipment (PPE) may place healthcare workers at an increased risk of exposure to COVID-19. The lack of access to personal protective equipment has been heavily reported in the press and social media platforms [16]. Several studies conducted in different areas of the world show that the factors affecting the knowledge, attitude, and practice of healthcare workers were age, sex, work experience, type of health facilities, training on standard precaution, and the profession of the healthcare workers.

In Ethiopian context, the government took numerous steps to identify, prevent, and control the pandemic. More efforts were made by the government and other stakeholders to increase the testing capacity, resulting in behavioural changes within the community [17]. There is paucity of literature on the practice of healthcare workers in the prevention and control measures of the COVID-19 pandemic. In addition, most of the studies conducted on Asian healthcare workers and medical students showed that they had inadequate knowledge regarding the prevention practices of COVID-19. However, they had a positive attitude towards COVID-19 prevention practices [18].

Healthcare workers' knowledge about the disease directly affects early diagnosis and giving appropriate treatment, which may result in the rapid spread of the infection in the community. This means having enough knowledge about a disease can always affect an individual's attitudes and practices; on the other hand, improper attitudes and practices can increase the risk of the disease and result in death [19]. There is the fact that all healthcare professionals should have a good prevention and control practice; however, few studies conducted in some parts of Ethiopia revealed that a significant number of healthcare workers had insufficient knowledge, attitude, and practice about COVID-19 infection prevention. There is a limited published study conducted in Ethiopia about the factors associated with the knowledge, attitude, and practice of prevention of coronavirus disease, specifically for HCWs. And none of the previously published studies conducted in Ethiopia investigated this topic among HCWs in both private and governmental health facilities at a zonal level.

Although there are some studies conducted on COVID-19 in Ethiopia, it's still a public health problem and needs more comprehensive studies to alleviate the problem and to my knowledge, there is no published study conducted to assess the knowledge, attitude, and practice of healthcare workers about COVID-19 in the study setting. Therefore, the purpose of this study was to assess the level of practice, of prevention, and control measures of COVID-19 pandemic and the associated factors among healthcare workers in health facilities of the Horo Guduru Wollega Zone, West Ethiopia.

## 2. Materials and Methods

### 2.1. Ethical Consideration

To conduct the study, an ethical approval and supporting letter was obtained from the Institutional Review Board (IRB) of Jimma University Institute of Health with ref. No. JHRPG/89/21, and the Department of Epidemiology with ref. No. EPID/268/2021, respectively. Data collection was started after permission and the cooperation letter was written to all health facilities on which the study was carried out.

The study title, purpose, procedure and duration, and the possible risks and benefits of the study were clearly explained for the participants. Then, individual informed written consent was taken from the respondents and were assured of confidentiality by excluding their names during the period of data entry and analysis. They were informed well that they have full right to totally refuse to participate and/or withdraw from the study at any time if they have any difficulty.

### 2.2. Study Period

The study was conducted in the Horo Guduru Wollega Zone Health facilities from May to June 2021.

### 2.3. Study Design

Quantitative, facility-based cross-sectional study design was conducted from May to June 2021 using a self-administered structured questionnaire.

### 2.4. Population

#### 2.4.1. Source of Population

All healthcare workers who were working in public and private health facilities in the Horo Guduru Wollega Zone in 2021 were the source.

#### 2.4.2. Study Population

All randomly selected HCWs having direct contact with patients, in randomly selected health facilities in the Horo Guduru Wollega Zone, West Ethiopia were the study population.

### 2.5. Inclusion and Exclusion Criteria

#### 2.5.1. Inclusion Criteria

Healthcare workers who have direct contact with patients (i.e., physicians, nurses, health officers, laboratory technologists, pharmacists, anaesthetists, and midwives) and in selected health facilities were included in the study.

#### 2.5.2. Exclusion Criteria

Healthcare professionals who have no direct patient contact (who are on management positions and Health office workers) and supportive staff were excluded.

### 2.6. Sample Size Determination

The sample size was calculated separately for descriptive and analytical objectives and that which yields the maximum sample size was taken. Accordingly, significantly associated factors with the practice of HCWs from all related studies were reviewed. Among them, the availability of PPE (% outcome in the unexposed group 42.8 and AOR 1.96.) with the prevention practice of COVID-19 was taken from Tsegaye D. [20]. Then, using Epi-info™ version 7 StatCalc. software by considering 80% power, 95% CI, and exposed to the unexposed ratio of 1 : 1, we got 304 and afterwards added 10% nonresponse rate, making the final sample size 334.

### 2.7. Sampling Procedure

A stratified two-stage sampling technique was used. The health facilities were stratified to hospitals, health centers, and private clinics. After stratification of the health facilities, a two-stage sampling technique was used. The first stage involves the selection of the health facilities from each stratum using the Simple Random Sampling (SRS) technique. The second stage involves the selection of eligible healthcare workers in each strata using the simple random sampling technique till the proportionally allocated sample size to each health facility. There are 3 hospitals (1 general and 2 primary), 51 health centers, and 44 private clinics (5 mediums and 41 primary clinics) in the zone. From these health facilities, 2 hospitals (1 general and 1 primary), 14 health centers, and 5 mediums (no higher clinics) were randomly selected. After the selection of these health facilities, only eligible healthcare workers were randomly selected from each health facility using the principle of proportional allocation. Finally, a total of 334 (hospitals = 136, health centers = 178, and private clinics = 20) healthcare workers were selected.

### 2.8. Data Collection Methods

#### 2.8.1. Data Collection Tool

A structured self-administered questionnaire was used to collect the data from the study participants. The tool was adapted and modified into the local context from previously published articles conducted in Ethiopia [21, 22] and it was prepared in the English language. The questions contains socio-demographic characteristics, the knowledge, attitude, and practice of coronavirus prevention, and also health service facilities and individual health workers' related factors. The questionnaire comprised a total of 58 questions. The first part has questions regarding the socio-demographic characteristics of the participants, which contain 11 questions; the second part is about knowledge regarding coronavirus, which contain 19 questions; the third part is about the attitudes of healthcare workers regarding coronavirus, which contain 15 questions; and the fourth part has questions about the practices of healthcare workers regarding coronavirus, which contain 13 questions. Questions of the health facility and individual health workers' related factors were merged with the socio-demographic and practice questions.

The knowledge questions include the respondents' knowledge about the cause, transmission route, clinical symptoms, and prevention and control measures of COVID-19. Respondents were given response options and the correct response was assigned 1 point, while an incorrect or “I do not know” response to a question was assigned 0 points. There are two multiple response questions, and each options carries one point. Each respondent achieves between 2 and 26 score points. A higher score indicates better knowledge of COVID-19. The attitudes questions include the cause of COVID-19, ways of transmission, confidence of the respondent on COVID-19 prevention methods, and confidence on the government to contain the spreading of the virus. Respondents were given a 5-Likert scale “Strongly Agree,” “Agree,” “Neutral,” “Disagree,” And “Strongly Disagree” response options and responses were assigned 5, 4, 3, 2, and 1 points, respectively. Each question carries five points and each respondent achieves between 13 and 75 score points. The practice questions include the respondents' practice about the prevention and control measures and reasons of not using COVID-19 prevention and control measures. Respondents were given three response options: “No,” “Sometimes,” and “Every time” and were coded as 0, 1, and 2, respectively. If the participants are not using COVID-19 prevention and control measures, they were asked additional questions to know their reason of not using. Each respondent achieves between 0 and 14 score points.

#### 2.8.2. Data Collectors and Data Collection Procedure

The data were collected by a total of seven data collection facilitators who have a minimum of diploma in nursing/midwifery. Two supervisors with BSc in Public Health or Nursing were selected. Data collection facilitators and supervisors were trained for one day. After the one-day training, the questionnaires were distributed to the data collection facilitators through the supervisors at the respective sites. Then, the data collection facilitators secured the written informed consent and administered the prepared questionnaires to the study participants and collected the filled questionnaires from the participants. Necessary COVID-19 preventive and control measures were applied in the entire process of the study.

### 2.9. Data Processing

The collected data were checked for completeness and internal consistency. It was then coded and entered into EpiData version 3.1 software packages and exported to Statistical Package for Social Science (SPSS) version 21 software for analysis.

### 2.10. Statistical Analysis

The descriptive analysis of data was done using numerical summary measures and the data were presented using frequency tables, figures, and graphs. Knowledge, attitudes, and practices' scores were calculated to give the overall knowledge, attitude, and practice score. Bivariable analysis was done to test for an association between the dependent and independent variables. All important independent variables were entered to multivariable binary logistic regression to identify the associated factors between the categorical dependent variable (practice) and the independent variables. Model fitness and multicollinearity were checked using the Hosmer-Lemeshow goodness of fit test and variance inflation factor (VIF). The results of logistic regression were reported as adjusted odds ratio (AOR). Those variables with a *p* value less than 0.05 with 95% confidence interval were considered as significantly associated with the outcome variable.

### 2.11. Data Quality Management

The data collection facilitators and supervisors were provided with intensive training on the objective of the study, contents of the questionnaires, and how to maintain confidentiality and privacy of the study subjects. Pretest was conducted two weeks prior to the actual data collection period on 17 healthcare workers and necessary modification was made on the questionnaires. The collection of data was checked by principal investigators on a daily basis for any incompleteness and/or inconsistency. Each questionnaire is identified by the ID given for it.

## 3. Results

A total of 325 healthcare workers participated in this study, which yields a response rate of 97.3%. Four HCWs were not willing to participate in the study due to different reasons and five HCWs submitted incomplete questionnaires.

### 3.1. Sociodemographic Characteristics

As presented in Table 1, 276 (84.9%), the majority of the participants' age was between 25 and 30 years old with a mean (±SD) age of 27.84 (±2.575) years. The majority, 196 (60.3%), of the respondents were males and more than two-third of the participants were followers of the protestant religion. More than two-third, 224 (68.9%), of the respondents were married. Most respondents, 137 (42.2%), were nurses, and 53 (16%) were midwifes. Half of the respondents were from government health centers and diploma holders. The majority, 227 (69.8%), of the respondents have service years less than five years. Thirty-seven (11.4%) participants had a preexisting medical condition (Table 1).

### 3.2. Practice of Healthcare Workers on the Prevention and Control Measures of COVID-19 Infection

As presented in Table 2, this study showed that 208 (64%; 95% CI: (58.8%, 69.2%)) respondents had good practice towards COVID-19 and its prevention, and the mean (±SD) practice score was 7.63 ± 2.45. Based on the result, 114 (35.1%) respondents were using a facemask every time and 97 (29.8%) were using gloves for every patient. Among the participating healthcare workers, only 46 (14.2%) practiced hand washing (among those only 47.2% use soap to wash their hands) and 89 (27.4%) used antiseptics/sanitizer every time. One hundred three (31.7%) healthcare workers refrained from touching their face and only 16.3% of the respondents were practicing social distancing every time. 223 (68.6%) healthcare workers were still practicing handshaking sometimes and every time (Table 2).

#### 3.2.1. Reasons for Not Practicing PPE

**Figure 1:**
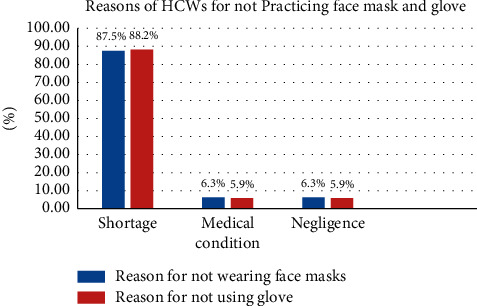
Reasons of the HCWs for not using facemasks and gloves in the health facilities.

**Figure 2:**
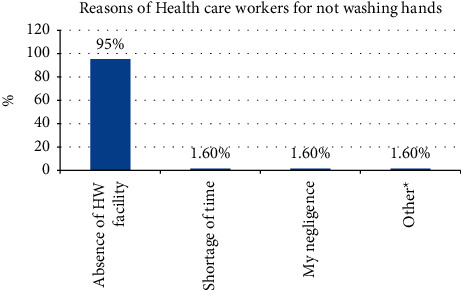
Reasons of the HCWs for not washing hands in the health facilities.

The majority (87.5%) of the HCWs who did not wear facemasks stated that shortage was a reason for not using it. The majority (88.24%) of the HCWS stated shortage as a reason for not using gloves. The majority (95.29%) of the HCWs, from those who did not wash their hands, claimed the absence of a hand washing facility as a reason for not washing their hands (Figures 1 and 2).

### 3.3. Knowledge of the Healthcare Workers about COVID-19 Infection

**Figure 3:**
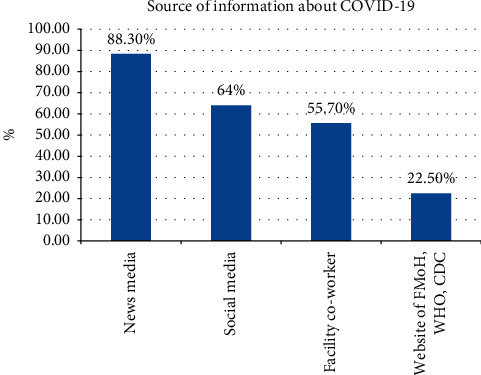
Source of information about COVID-19 for health workers.

As presented in Table 3 and Figure 3, this study revealed that 266 (81.8%; 95% CI: (77.6%, 86.1%)) respondents demonstrated self-reported sufficient knowledge towards COVID-19 and the mean (±SD) knowledge score was 22.39 (±2.27). Three hundred and eleven (95.7%) respondents know that COVID-19 is a viral disease and 275 (84.6%) respondents responded that asymptomatic persons with COVID-19 can transmit the disease to other people. The respondents' correct answer rates on the transmission of COVID-19 were 90.5%. Two hundred and ninety-eight (91.7%) participants responded that COVID-19 could be fatal and 312 (96.0%) of them think the risk of infection and death from COVID-19 are higher among patients with underlying chronic illnesses. About 87.1% of the participants said that COVID-19 has an effective vaccine, whereas 88.6% and 92% of the participants said that COVID-19 has no specific treatment and symptomatic and supportive care is the current treatment for COVID-19 as of today, respectively. Less than half (44.3%) of the participants know the national/regional COVID-19 response phone address (Table 3). News media and social media were major sources of information (Figure 3).

### 3.4. Attitude of the Healthcare Workers towards COVID-19 Infection

As presented in Table 4, this study found that 243 (74.8%; 95% CI: (70.0%, 79.5%)) participants have a favourable attitude towards COVID-19 and the mean (±SD) attitude score was 57.04 (±6.0). The majority, 316 (97.2%), of the respondents agreed that COVID-19 is a seriously dangerous disease and 228 (70.1%) believed that COVID-19 will finally be successfully controlled. Three hundred and twenty (98.4%) and 321 (98.8%) respondents agree that hand hygiene and wearing facemasks is important in controlling the spread of COVID-19, respectively. One hundred and eighty-eight (57.8%) respondents think they may probably be infected with COVID-19. On the other hand, only 182 (56%) accept isolation in health facilities if they get infected with COVID-19. About 221 (68%) respondents believed that taking hot drinks prevents COVID-19 infection, 42 (13%) said COVID-19 will not spread in hot climate areas, and 67 (20.7%) believed herbal medications will cure COVID-19. Among the study participants, only 95 (29.2%) are confident enough to treat a confirmed case of COVID-19 if encountered. However, 98 (30.2%) providers have no confidence that Ethiopia can win the battle against the COVID-19 virus (Table 4).

### 3.5. Factors Associated with the Practice of Healthcare Workers on the Prevention and Control Measures of COVID-19 Infection

All-important independent variables: sex, age, marital status, the profession of the respondent, service year, the type of health institution, average monthly income, the current working unit of the respondent, educational level, medical condition, the training status of the respondents, knowledge category, and attitude category were entered to the multivariable binary logistic regression analysis by the “ENTER METHOD.” Model fitness was checked by the Hosmer-Lemeshow test. Accordingly, the result was 0.195 for factors associated with the practice of the HCWs and then, the multivariable binary logistic regression analysis showed that the type of health facility, the current working unit, being trained on standard precaution, and knowledge category were associated with a good preventive practice. The odds of good practice was reduced by 74% (AOR = 0.26; 95% CI: (0.13, 0.52)) for healthcare workers working in government health centers compared to those working in government hospitals. The odds of good practice were 4 times (AOR = 4.05; 95% CI: (1.22, 13.42)) higher among healthcare workers working in laboratories compared with healthcare workers working in the OPD/Emergency room. The odds of good practice were 2.73 times (AOR = 2.73; 95% CI: (1.35, 5.53)) higher among healthcare workers who have sufficient knowledge compared with healthcare workers who have insufficient knowledge. The odds of having good practice on COVID-19 were reduced by 66% (AOR: 0.34; 95% CI (0.19, 0.60)) among healthcare workers who were not trained on standard precaution compared with trained healthcare workers (Table 5).

## 4. Discussion

Although the Ethiopian government and other stakeholders took numerous steps to identify, prevent, and control the pandemic and more efforts are being made by government to increase the testing capacity that would result in behavioural changes within the community [17], this study found that 64% of the respondents had good practices of prevention and control measures of COVID-19. This finding is supported by reports from a study in Northern Ethiopia among nurses. 67% had good infection prevention practice towards the COVID-19 infection [22]. The finding is lower than the study conducted in Henan, China 89.7%, Pakistan 88.7%, Saudi Arabia 81.9%, Nepal 78.9%, and a finding from Makerere University Teaching Hospitals, Uganda 74% [2, 23–26]. The possible reason for the current low practice might be due to the variation in the cut-off point which is used to determine the outcome variable and the variation in the number and type of healthcare facilities included in these studies. Additionally, this study found that 52% of the healthcare workers had taken any form of training or refresher courses. This finding was very high compared with a study from Northwest Ethiopia and Libya, which shows that less than 24.8% and 7% of the healthcare workers had attended formal training, discussions, and lectures about COVID-19 [22, 27]. This much difference might be due to the variation in the study participants, the study period, and differences in the study setting; this study included participants from hospitals, health centers, and private clinics, whereas the previous study was hospital and health center based.

The study further showed that the majority (81.8%) of the health workers that participated in the study had self-reported sufficient knowledge about COVID-19. The finding is consistent with the findings of studies in Saudi Arabia 81.3% and a study conducted among nurses at the Gondar Zone 84.9% [24, 28]. However, the finding of the current study is higher than the finding of the National Survey conducted among the healthcare workers in Nepal 76%, at the Makerere University Teaching Hospitals (MUTHs) in Uganda 69%, and a survey conducted at northwest Ethiopia 73.8% [22, 25, 26]. This high percentage of knowledge about COVID-19 among healthcare workers might be due to the prolonged exposure to relevant information since its global headline of news on the media and public. Another reason could be the effort of the government and media in providing sufficient information starting from the time of the outbreak. The main sources of information in this study were News media (TV and/or radio) and social media, 88.3% and 64%, respectively, whereas those of the study by Huynh G. were social media and the Ministry of Health website, 91.1% and 82.6%, respectively [29]. The possible justification for the difference could be the difference in the study population, the study period, and infrastructures such as electricity and telecommunication.

In this study, the overall favourable attitude towards COVID-19 was 74.8% among the healthcare workers working in the Horo Guduru Zone. This finding was higher than a study conducted in Nepal, 54.7%, in Sub-Saharan Africa, at the Makerere University Teaching Hospitals (MUTHs) in Uganda, and a study conducted among nurses at the Gondar Zone, Somali region, Ethiopia which brought out 21%, 63.3%, and 45.2% of the participants had a positive attitude [25, 26, 28, 30]. However, this finding was lower than the study conducted on Vietnamese healthcare workers 90.0%, a study in Saudi Arabia 81.3%, and a multicenter study from Ethiopia 94.7% [21, 24, 29]. This difference may be due to the variation in the cut-off point which is used to determine the outcome variable, variation in the type and number of healthcare facilities included in these studies, and variation in the country's healthcare system, information, protection, and support which will affect the providers' attitude towards the disease. Previous studies have shown that during the time of such disease outbreaks, healthcare workers are prone to mental health diseases [31] and in this study, 57.8% of the healthcare workers fear that they may probably be infected with COVID-19, and 11.7% of the providers responded that they would not accept isolation in isolation centers if they were infected and 32.3% responded neutral. People under isolation might experience social stigma, be considered a public health threat, self-blame, have a sense of being punished or maybe discriminated, which can result in negative consequences on peoples' cooperation for infection control [32].

This study showed that the odds of good practice was 74% lower among healthcare workers working in government health centers and 62% lower among healthcare workers working in private clinics compared with healthcare workers working in government hospitals. Justification of this may be that healthcare workers working in hospitals may have the highest skilled manpower as compared to health centers and private clinics, which are mostly composed of diploma holders. Moreover, hospitals are also organized with good supply of personal protective equipment. Generally, among the assessed factors, insufficient knowledge, being untrained on infection prevention and control measures, and shortage of personal protective equipment were the perceived barriers to infection prevention practices. These findings were in line with a study conducted in the Ilu Aba Bor and Buno Bedelle Zones [20].

## 5. Conclusion

In conclusion, this study has illuminated the current magnitude of practice of prevention and control measures of healthcare workers on COVID-19 among the Horo Guduru Wollega Zone. Despite the fact that all HCWs should be knowledgeable, have favourable attitudes, and good prevention and control practice on COVID-19 infection, in the present study, the knowledge level, attitudes, and practices of HCWs on COVID-19 infection prevention and control measures were insufficient because 18.2%, 25.2%, and 36% of the healthcare workers did not have sufficient knowledge, favourable attitudes, and good practices of prevention and control measures, respectively. This study found that being health center workers, being trained, and having sufficient knowledge were associated with the outcome variable, i.e., good practice of infection prevention and control measures.

## 6. Recommendations

Based on the study findings, the following recommendations were forwarded to the policy makers, national, regional, and zonal health bureaus, for healthcare workers, and researchers. Policy makers need to expand sustainable infection control and prevention strategies for healthcare professionals and the community at large. The magnitude of practice of prevention and control measures of COVID-19 infection indicates the need for more work; thus, the regional health bureau and zonal health department need to consider the importance of trainings and refresher courses on standard precaution so as to protect them from acquiring the infection. Additionally, continuous provision of PPE for all healthcare workers is vital. Healthcare workers also need to adhere to the principles of standard precautions along with enhancing their awareness on the proper practice and take commitment to protect themselves from this virus. It is better if a qualitative study is conducted on COVID-19, considering the barriers that contribute for the quick distributions and the reasons for the reduction of the preventive practice toward this pandemic disease. Finally, further investigation is needed for vaccine coverage with laboratory confirmation of the vaccination status of HCWs.

### 6.1. Limitations of the Study

Although this study has wide area coverage, it has some limitations. The response was self-reported; because of this, recall bias could happen and this may lead to the over or underestimation of preventive practice. Causal inferences may not be established due to the study design used (cross-sectional). The study was conducted in a single zone with a lower number of detected COVID-19 infections than other zones, which might have affected the results. The other limitation of this study is it did not include the vaccination status of the healthcare workers.

## Figures and Tables

**Table 1:** Sociodemographic characteristics of the healthcare workers in the health facilities of the Horo Guduru Wollega Zone, West Ethiopia, 2021 (*n* = 325).

Variables	Category	Frequency	Percent
Age classification	<25 year	47	14.5
	25–35 year	276	84.9
	≥35 year	2	0.6

Sex	Male	196	60.3
	Female	129	39.7

Religion	Orthodox	76	23.4
	Muslim	12	3.7
	Protestant	229	70.5
	Wakefeta	8	2.5

Marital status	Married	224	68.9
	Single	99	30.5
	Divorced	2	0.6

Profession	Nurse	137	42.2
	Midwife	53	16.3
	Health officer	30	9.2
	Laboratory technologist	43	13.2
	Pharmacist	32	9.8
	General practitioner	21	6.5
	Specialist	9	2.8

Type of health institution	Government hospital	137	42.2
	Government health center	163	50.2
	Private clinic	25	7.7

Current working unit	Out-patient department	53	16.3
	Emergency room	35	10.8
	Inpatient	42	12.9
	Maternal and child health	60	18.5
	Laboratory	41	12.6
	Pharmacy	32	9.8
	Others	62	19.1

Educational level	Diploma	171	52.6
	Bsc	142	43.7
	Msc/MPH	4	1.2
	Specialty	8	2.5

Service year	<5 year	227	69.8
	6–10 year	90	27.7
	>10 year	8	2.5

Presence of preexisting medical condition	Yes	37	11.4
	No	288	88.6

**Table 2:** Practice of the healthcare workers about COVID-19 infection in the health facilities of the Horo Guduru Wollega Zone, West Ethiopia, 2021 (*n* = 325).

Variables	No	Sometimes	Every time
Do you use face masks for COVID-19 prevention?	16 (4.9%)	195 (60.0%)	114 (35.1%)
Do you use gloves for prevention throughout your practice in healthcare facilities?	34 (10.5%)	194 (59.7%)	97 (29.8%)
Do you wash your hands for prevention throughout your practice in HC facilities?	162 (49.8%)	117 (36.0%)	46 (14.2%)
Are you using soap to wash hands?	25 (15.3%)	61 (37.4%)	77 (47.2%)
Do you use antiseptics/sanitizer?	18 (5.5%)	218 (67.1%)	89 (27.4%)
Do you refrain from touching your face?	49 (15.1%)	173 (53.2%)	103 (31.7%)
Are you practicing social distancing?	63 (19.4%)	209 (64.3%)	53 (16.3%)
Are you practicing handshaking?	102 (31.4%)	167 (51.4%)	56 (17.2%)

**Table 3:** Knowledge of the healthcare workers about COVID-19 infection in the health facilities of the Horo Guduru Wollega Zone, West Ethiopia, 2021 (*n* = 325).

Variables	Yes	No	I do not know
COVID-19 is a viral infection?	311 (95.7%)	0 (0.0%)	14 (4.3%)
Asymptomatic persons with COVID-19 can transmit the disease to other people?	275 (84.6%)	24 (7.4%)	26 (8.0%)
Healthcare workers are at higher risk of infection?	312 (96.0%)	7 (2.2%)	6 (1.8%)
COVID-19 is transmitted via respiratory droplets?	294 (90.5%)	5 (1.5%)	26 (8.0%)
COVID-19 could be fatal?	298 (91.7%)	12 (3.7%)	15 (4.6%)
Risk of infection and death from COVID-19 are higher among patients with underlying chronic illnesses	312 (96.0%)	6 (1.8%)	7 (2.2%)
Is there an effective vaccine for COVID-19 prevention?	283 (87.1%)	19 (5.8%)	23 (7.1%)
Are antibiotics effective in preventing and treating COVID-19?	40 (12.3%)	266 (81.8%)	19 (5.8%)
Is there an effective medication for COVID-19 treatment as of today?	12 (3.7%)	288 (88.6%)	25 (7.7%)
Symptomatic and supportive care is the current treatment for COVID-19?	299 (92.0%)	12 (3.7%)	14 (4.3%)
Do you know the national/regional COVID-19 response phone address?	144 (44.3%)	41 (12.6%)	140 (43.1%)

**Table 4:** Attitude of the healthcare workers towards COVID-19 infection in the health facilities of the Horo Guduru Wollega Zone, West Ethiopia, 2021 (*n* = 325).

Variables	Strongly disagree	Disagree	Neutral	Agree	Strongly agree
Do you perceive COVID-19 as a dangerous disease?	2 (0.6%)	2 (0.6%)	5 (1.5%)	131 (40.3%)	185 (56.9%)
Do you believe that COVID-19 will finally be successfully controlled?	4 (1.2%)	30 (9.2%)	63 (19.4%)	108 (33.2%)	120 (36.9%)
Do you think all white people can transmit COVID-19?	169 (52.0%)	85 (26.2%)	40 (12.3%)	13 (4.0%)	18 (5.5%)
Do you think hand hygiene is important in controlling the spread COVID-19?	0 (0.0%)	1 (0.3%)	4 (1.2%)	123 (37.8%)	197 (60.6%)
Do you think wearing masks is important in controlling the spread COVID-19?	0 (0.0%)	0 (0.0%)	4 (1.2%)	79 (24.3%)	242 (74.5%)
Do you think you may probably get infected with COVID-19?	21 (6.5%)	66 (20.3%)	50 (15.4%)	110 (33.8%)	78 (24.0%)
If you get infected with COVID-19, will you accept isolation in health facilities?	3 (0.9%)	35 (10.8%)	105 (32.3%)	113 (34.8%)	69 (21.2%)
Do you fear that you may transmit COVID-19 to your family members?	7 (2.2%)	14 (4.3%)	99 (30.5%)	140 (43.1%)	65 (20.0%)
Do you think that every HCW working at a COVID-19 treatment center needs to be quarantined though asymptomatic?	3 (0.9%)	11 (3.4%)	62 (19.1%)	173 (53.2%)	76 (23.4%)
Do you think herbal medication can cure COVID-19?	48 (14.8%)	112 (34.5%)	98 (30.2%)	59 (18.2%)	8 (2.5%)
Do you think COVID-19 is a curse?	137 (42.2%)	63 (19.4%)	44 (13.5%)	55 (16.9%)	26 (8.0%)
Do think COVID-19 will spread in hot climate?	7 (2.2%)	35 (10.8%)	138 (42.5%)	86 (26.5%)	59 (18.2%)
Do you think taking hot drinks will prevent COVID-19	11 (3.4%)	24 (7.4%)	69 (21.2%)	136 (41.8%)	85 (26.2%)
Are you confident enough to treat a confirmed case of COVID-19 if you encountered?	38 (11.7%)	107 (32.9%)	85 (26.2%)	82 (25.2%)	13 (4.0%)
Do you have confidence that Ethiopia can win the battle against the COVID-19 virus?	20 (6.2%)	78 (24.0%)	117 (36.0%)	67 (20.6%)	43 (13.2%)

**Table 5:** Factors associated with the practice of the respondents on the prevention and control measures of COVID-19 in the health facilities of the Horo Guduru Wollega Zone, West Ethiopia, 2021 (*n* = 325).

Characteristics	Category	Practice category		COR (95%CI)	AOR (95%CI)
		Good practice	Poor practice		
Sex	Male	141 (43.4%)	55 (16.9%)	1	1
	Female	67 (20.6%)	62 (19.1%)	0.42 (0.27, 0.67)^^*∗*^^	0.95 (0.48, 1.88)

Age	≤25	37 (11.4%)	10 (3.1%)	1	1
	>25	171 (52.6%)	107 (32.9%)	0.43 (0.21, 0.91)^*∗*^	0.54 (0.21, 1.39)

Marital status	Married	131 (40.3%)	93 (28.6%)	1	1
	Single/divorced	77 (23.7%)	24 (7.4%)	2.3 (1.34, 3.87)^*∗*^	1.4 (0.67, 2.90)

Profession	Nurse	88 (27.1%)	49 (15.1%)	.1	1
	Midwife	26 (8.0%)	27 (8.3%)	0.54 (0.28, 1.02)	0.84 (0.30, 2.39)
	Other professions	94 (28.9%)	41 (12.6%)	0.25 (0.77, 2.12)	0.80 (0.34, 1.86)

Work experience	≤5	142 (43.7%)	85 (26.2%)	1	1
	>5	66 (20.3%)	32 (9.8%)	1.24 (0.75, 2.04)	1.5 (0.83, 2.71)

Type of health institution	Government hospital	109 (33.5%)	28 (8.6%)	1	1
	Government health center	84 (25.8%)	79 (24.3%)	0.27 (0.16, 0.46)^*∗*^	0.26 (0.13, 0.52)^*∗*^
	Private clinic	15 (4.6%)	10 (3.1%)	0.39 (0.16, 0.95)^*∗*^	0.38 (0.12, 1.16)

Monthly income	≤5000	81 (24.9%)	65 (20.0%)	1	1
	>5000	127 (39.1%)	52 (16.0%)	1.96 (1.24, 3.10)^*∗*^	1.6 (0.84, 3.00)

Current working unit	OPD/emergency room	55 (16.9%)	33 (10.2%)	1	1
	Inpatient	35 (10.8%)	7 (2.2%)	3 (1.20, 7.52)^*∗*^	1.86 (0.59, 5.80)
	MCH	30 (9.2%)	30 (9.2%)	0.60 (0.31, 1.17)	0.91 (0.32, 2.60)
	Laboratory	30 (9.2%)	11 (3.4%)	1.64 (0.73, 3.7)	4.05 (1.22, 13.42)^*∗*^
	Pharmacy	19 (5.8%)	13 (4.0%)	0.88 (0.38, 2.01)	1.9 (0.58, 6.18)
	Others	39 (12.0%)	23 (7.1%)	1.02 (0.52, 2.00)	1.3 (0.56, 2.96)

Educational level	Diploma	90 (27.7%)	81 (24.9%)	1	1
	BSc and above	118 (36.3%)	36 (11.1%)	2.95 (1.83, 4.76)^*∗*^	1.25 (0.57, 2.74)

Did you have a preexisting medical condition?	Yes	24 (7.4%)	13 (4.0%)	1	1
	No	184 (56.6%)	104 (32.0%)	0.96 (0.47, 1.96)	1.15 (0.49, 2.73)

Trained on standard precaution?	Yes	134 (41.2%)	35 (10.8%)	1	1
	No	74 (22.8%)	82 (25.2%)	0.24 (0.15, 0.38)^*∗*^	0.34 (0.19, 0.60)^*∗*^

Knowledge category	Insufficient knowledge	23 (7.1%)	36 (11.1%)	1	1
	Sufficient knowledge	185 (56.9%)	81 (24.9%)	3.58 (1.99, 6.42)^*∗*^	2.73 (1.35, 5.53)^*∗*^

Attitude category	Unfavourable attitude	41 (12.6%)	41 (12.6%)	1	1
	Favourable attitude	167 (51.4%)	76 (23.4%)	2.20 (1.32, 3.66)^*∗*^	1.67 (0.86, 3.24)

^*∗*^Statistically significant variables at *p* < 0.05.

## Data Availability

The datasets used for analysis are available from the corresponding author on reasonable request.

## Abbreviations

AOR: Adjusted odds ratio
CDC: Center for Disease Control
CI: Confidence interval
COR: Crude odd ratio
COVID-19: Coronavirus disease 2019
CSA: Central Statistical Agency
HCWs: Healthcare workers
OR: Odds ratio
PAHO: Pan American Health Organization
PHP: Primary healthcare professional
PPE: Personal protective equipment
SARS: Severe acute respiratory syndrome
SPSS: Statistical Package for Social Science
SRS: Simple random sampling
VIF: Variance inflation factor
WHO: World Health Organization.
